# Long-term follow-up of predominantly Asian patients with relapsed/refractory *FLT3*-mutated acute myeloid leukemia in the phase 3 COMMODORE trial

**DOI:** 10.1007/s00277-026-06762-2

**Published:** 2026-01-12

**Authors:** Bin Jiang, Jian Li, Ligen Liu, Xin Du, Hao Jiang, Jianda Hu, Xiaoxi Zeng, Taishi Sakatani, Masanori Kosako, Yaru Deng, Vladimir Ivanov, Sergey Bondarenko, Lily Wong Lee Lee, Archrob Khuhapinant, Elena Martynova, Nahla Hasabou, Jamie Jung-Hee An, Jianxiang Wang

**Affiliations:** 1 Department of Hematology, Peking University International Hospital, Beijing, China; 2 Department of Hematology, Peking Union Medical College Hospital, Beijing, China; 3 Department of Hematology, Tongren Hospital Shanghai Jiao Tong University School of Medicine, Shanghai, China; 4 Department of Hematology, Guangdong Provincial People’s Hospital, Guangzhou, China; 5 Department of Hematology, Peking University People’s Hospital, Beijing, China; 6 Fujian Institute of Hematology, Fujian Medical University Union Hospital, Fuzhou, Fujian China; 7 The Second Affiliated Hospital of Fujian Medical University, Quanzhou, China; 8 Astellas China Investment Co, Ltd., Beijing, China; 9 Astellas Pharma, Inc., Tokyo, Japan; 10 Personalized Medicine Center, Almazov National Medical Research Center, St. Petersburg, Russia; 11 Department of Bone Marrow Transplantation of Adults, RM Gorbacheva Research Institute, Pavlov University, St. Petersburg, Russia; 12 Department of Hematology, Queen Elizabeth Hospital, Kota Kinabalu, Sabah Malaysia; 13 Division of Hematology, Department of Medicine, Faculty of Medicine Siriraj Hospital, Mahidol University, Bangkok, Thailand; 14 Department of Hematology and Chemotherapy, Krasnoyarsk Regional Clinical Hospital, Krasnoyarsk, Russia; 15 Astellas Pharma, Inc., Northbrook, IL USA; 16 Astellas Pharma, Inc., Singapore, Singapore; 17 State Key Laboratory of Experimental Hematology, National Clinical Research Center for Blood Diseases, Institute of Hematology and Blood Diseases Hospital, Chinese Academy of Medical Sciences and Peking Union Medical College, Tianjin, China

**Keywords:** Acute myeloid leukemia, Gilteritinib, FLT3, Phase 3, Clinical trials

## Abstract

To evaluate the long-term efficacy and safety of gilteritinib compared with salvage chemotherapy (SC) in patients with relapsed/refractory *FMS*-like tyrosine kinase 3 (*FLT3*)-mutated acute myeloid leukemia (AML). In the phase 3 COMMODORE (NCT03182244) trial, patients with relapsed/refractory *FLT3*-mutated AML from China, Russia, Singapore, Thailand, and Malaysia were randomized to gilteritinib (120 mg/day) or SC. The long-term follow-up included assessments every 3 months for a maximum of 3 years from the end-of-treatment visit. The primary endpoint was overall survival (OS). Secondary endpoints included event-free survival (EFS), complete remission (CR) rate, hematopoietic stem cell transplantation (HSCT) rate, and transfusion maintenance and conversion rates. Overall, 276 patients (gilteritinib, *n* = 137; SC, *n* = 139) completed the long-term follow-up. Most (88.0%) patients were Asian. The median (95% confidence interval [CI]) OS was longer with gilteritinib versus SC (10.3 [8.8, 12.7] vs 5.4 [4.1, 8.1] months, respectively; hazard ratio [HR; 95% CI], 0.612 [0.451, 0.832]), with a median follow-up of 34.6 months. The median (95% CI) EFS was longer with gilteritinib versus SC (2.1 [< 0.1, 3.2] vs 0.6 [0.2, 1.2] months, respectively; HR [95% CI], 0.589 [0.438, 0.792]). The CR rate was 20.4% and 11.5% in the gilteritinib and SC arms, respectively. During the entire study period, 22.6% and 7.9% of patients in the gilteritinib and SC arms underwent HSCT, respectively; 18.2% of patients in the gilteritinib arm received on-study HSCT. No new safety concerns were identified. Long-term gilteritinib treatment improved clinical outcomes compared with SC and was well-tolerated in a predominantly Asian population with relapsed/refractory *FLT3*-mutated AML.

**Supplementary Information:**

The online version contains supplementary material available at 10.1007/s00277-026-06762-2.

## Introduction

Advances in genomic profiling of acute myeloid leukemia (AML) have led to the development of targeted therapies and more treatment options for patients with relapsed/refractory (R/R) AML [1]. One of the most common AML mutations is the *FMS*-like tyrosine kinase 3 (*FLT3*) receptor gene with *FLT3* internal tandem duplications (*FLT3*-ITD) and point mutations in the tyrosine kinase domain (*FLT3*-TKD), which occurs in 18.9% to 24.9% [2–4] and 5.0% to 6.2% [4, 5] of newly diagnosed adult patients with AML, respectively. In addition, AML has significant clonal evolution, and mutations can be gained or lost during disease progression [6]. Patients with R/R *FLT3-*mutated (mut+) AML have poorer clinical outcomes compared with patients without *FLT3* mutations [7]; thus, additional treatment options are needed for this subset of patients.

FLT3-inhibiting agents have demonstrated improved survival and response rates in patients with R/R *FLT3*^mut+^ AML compared with salvage chemotherapy [8–10]. Gilteritinib, a second-generation FLT3 inhibitor that is effective against both *FLT3*-ITD and *FLT3*-TKD [11, 12], has received global regulatory approval, including in Europe [13], the United States [14], Japan [15], and China [16], for the treatment of R/R *FLT3*^mut+^ AML.

In Asia, the incidence of AML has increased in recent years [17]. Simultaneously, lack of access to newly approved treatments, novel therapies, clinical trials, and next-generation sequencing are considered challenges for patients with AML in the Asia-Pacific region [18] and may result in many patients going untreated. Consequently, there is a lack of data available on the long-term efficacy and safety of gilteritinib treatment in patients from Asia with R/R *FLT3*^mut+^AML. The phase 3 ADMIRAL trial evaluated the efficacy and safety of gilteritinib versus salvage chemotherapy in a global patient population with R/R *FLT3*^mut+^ AML [19]. Currently, the only published long-term follow-up data for gilteritinib are from the ADMIRAL trial [20], while randomized data in patients from Asia are restricted to a subgroup analysis of a small number of Japanese patients [21]. However, this analysis had a limited patient follow-up. In addition, there is evidence of gilteritinib being used as a bridge to hematopoietic stem cell transplantation (HSCT) [22], but published clinical data on the use of gilteritinib as post-HSCT maintenance therapy in the R/R setting remains scarce.

The COMMODORE trial evaluated the efficacy and safety of gilteritinib versus salvage chemotherapy in a predominantly Asian population of patients with R/R *FLT3*^mut+^ AML. In the primary analysis, with a median follow-up of 10.3 months, gilteritinib treatment resulted in significantly longer median overall survival (OS; 9.6 months) compared with salvage chemotherapy (5.0 months; hazard ratio [HR], 0.566; 95% confidence interval [CI], 0.392, 0.818; *P* = 0.00211). A regional subgroup analysis of the COMMODORE trial revealed that the effects of gilteritinib compared with salvage chemotherapy were maintained in separate cohorts of patients from China, Southeast Asia, and Russia [23]. Further characterization of gilteritinib’s long-term efficacy and safety in Asian patients is still needed. Here, we present the results of the long-term follow-up of the COMMODORE trial and results of an exploratory analysis of patients who underwent HSCT during the study period and resumed gilteritinib as post-HSCT maintenance treatment.

## Methods

### Study design

COMMODORE (NCT03182244) was a phase 3, open-label, multicenter, randomized study in patients with R/R *FLT3*^mut+^ AML. The study design and patient inclusion and exclusion criteria have been described in detail in Wang et al. 2024 [9]. Briefly, patients with R/R *FLT3*^mut+^ AML from 48 centers in China, Russia, Singapore, Thailand, and Malaysia were randomized 1:1 to gilteritinib (120 mg per day) or salvage chemotherapy (Supplementary Fig. 1). In the primary analysis, patients were followed from study initiation on October 25, 2017, until the interim analysis cut-off date of June 30, 2022 [9]; here, patients were followed beyond those 2.7 years until the date of last evaluation on December 25, 2023. Before randomization, a chemotherapy regimen was preselected by investigators for each patient from either low-dose cytarabine (LoDAC); mitoxantrone, etoposide, and intermediate-dose cytarabine (MEC); or fludarabine, high-dose cytarabine, and granulocyte colony-stimulating factor (FLAG). Patients received treatment over continuous 28-day cycles or as per their institutional guidelines for chemotherapy, and those who received gilteritinib or LoDAC continued treatment until treatment discontinuation criteria were met. Patients who received MEC or FLAG received 1 cycle of therapy and were assessed for response on or after Day 15 per institutional guidelines, with treatment decisions based on bone marrow cellularity. Patients in the gilteritinib arm who had an identified donor and achieved a response allowing HSCT could have undergone HSCT without leaving the study. Patients who underwent HSCT and met prespecified clinical criteria were able to resume gilteritinib treatment after HSCT. After treatment discontinuation, patients entered long-term follow-up for up to 3 years from the patient’s end-of-treatment visit.

## Ethics

The trial protocol was approved by institutional review boards or independent ethics committees at participating sites. This trial was conducted in accordance with the study protocol, the International Council on Harmonisation of Technical Requirements for Pharmaceuticals for Human Use guideline, the Declaration of Helsinki, and applicable regulations/guidelines that govern clinical study conduct and ethical principles. Written and signed informed consent was obtained from all patients or their guardian or legal representative prior to screening.

## Endpoints

The primary endpoint was OS. Secondary endpoints included: event-free survival (EFS), defined as the time from the date of randomization until the earliest date of relapse, treatment failure, death from any cause, reported off-treatment relapse, or the start of new AML therapy (excluding subsequent HSCT); complete remission (CR) rate; composite CR (CRc) rate; HSCT rate; transfusion conversion and maintenance rates (gilteritinib arm only); and safety. Response to treatment was assessed according to modified International Working Group criteria [24], as was done in the ADMIRAL trial [10], and is presented in Supplementary Table 1. Treatment failure was defined as failure to achieve CRc by the end of the treatment period. Adverse events of special safety interest (AESI) included anaphylactic reactions, cardiac failure, increased creatine phosphokinase, diarrhea, differentiation syndrome, gastrointestinal obstruction, gastrointestinal perforation, increased liver transaminase, pancreatitis, pericarditis/pericardial effusion, posterior reversible encephalopathy syndrome, QT prolongation, and teratogenicity and embryo-fetal deaths.

## Statistical analyses

This study had 1 planned interim analysis and an end-of-study analysis. The interim analysis (data cut-off date of June 30, 2020) was considered the primary analysis by the independent data monitoring committee and previously presented [9]; the prespecified end-of-study analysis covered the complete trial period. Efficacy analyses were performed on the intention-to-treat (ITT) population, which included all randomized patients, and safety analyses were performed on the safety analysis set (SAF), which included all patients who received at least 1 dose of study treatment. The Kaplan-Meier method was used to estimate OS and EFS and is reported with a corresponding 95% CI. The HR and 95% CI of the treatment effect were calculated with a stratified Cox proportional hazard model, and a 2-sided *P* value was calculated with a stratified log-rank test. The stratification factors were a patient’s response to first-line therapy and preselected salvage chemotherapy. The CR and CRc rates were analyzed using a stratified Cochran-Mantel-Haenszel test. As the primary analysis was considered the final analysis, the prespecified hierarchical testing procedure to maintain the overall 2-sided type 1 error rate at 0.05 was not applied, and all statistical tests of treatment effects should be considered nominal. One exploratory post hoc OS landmark analysis was conducted starting from 60 days post-HSCT based on treatment resumption, regardless of whether it occurred within 60 days post-HSCT or after, in patients in the gilteritinib arm who underwent HSCT and did not experience relapse within 60 days post-HSCT. Adverse events were summarized by the National Cancer Institute Common Terminology Criteria for Adverse Events v4.03. All data processing, summarization, and analyses were performed using SAS^®^ Version 9.4 or higher on Red Hat Enterprise Linux.

## Results

### Patient disposition

At the study completion date (date of last evaluation) of December 25, 2023, 276 patients had been randomly assigned to receive gilteritinib (137 patients) or salvage chemotherapy (139 patients) and were included in the ITT, which included 42 additional patients who were enrolled after the primary analysis [9]. The SAF included 253 patients (gilteritinib, *n* = 134; salvage chemotherapy, *n* = 119). Patients were allowed to switch from salvage chemotherapy to gilteritinib after the interim analysis, but none did. The median follow-up was 34.6 months, which is 24.3 months longer compared with the primary analysis (10.3 months) [9].

**Fig. 1:**
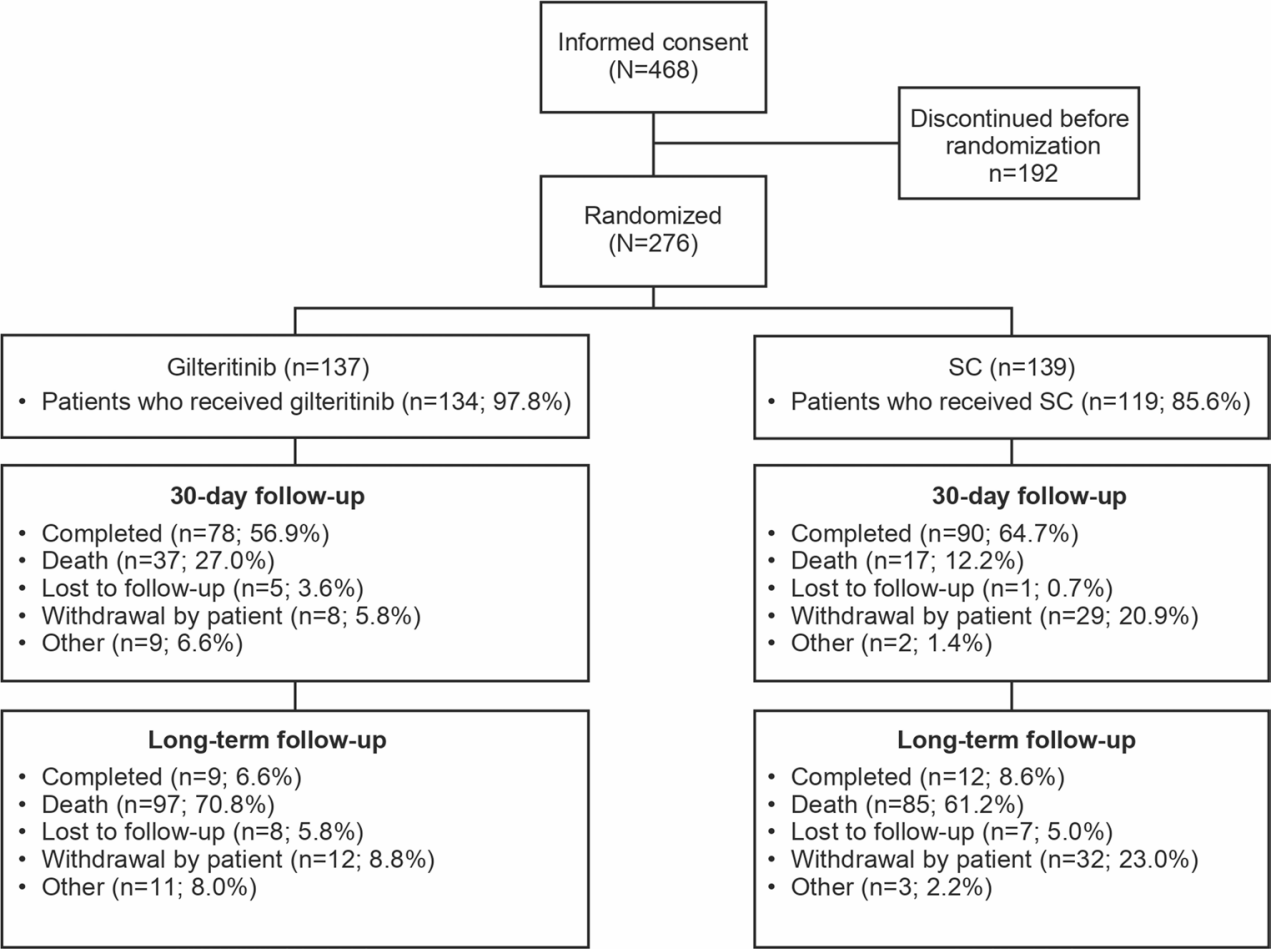
Patient disposition. Dispositions at the 30-day follow-up and long-term follow-up reflect patient status at this assessment date. Patients may have had different dispositions at the different assessments. SC, salvage chemotherapy

Most patients (65.9% [182/276]) were from China, followed by Russia (12.0% [33/276]). All patients ended their study treatment, including 23 patients who did not receive gilteritinib or salvage chemotherapy. The most frequent end-of-treatment reason was disease relapse (29.9% [41/137]) in the gilteritinib arm and lack of efficacy (30.9% [43/139]) in the salvage chemotherapy arm (Supplementary Table 2). In the gilteritinib arm, 56.9% (78/137) of patients completed the 30-day follow-up, and 64.7% (90/139) of patients in the salvage chemotherapy arm completed the 30-day follow-up (Fig. 1). The long-term follow-up was completed by 6.6% (9/137) of patients in the gilteritinib arm and 8.6% (12/139) of patients in the salvage chemotherapy arm. During the long-term follow-up, 8.8% (12/137) and 23.0% (32/139) patients in the gilteritinib and salvage chemotherapy arms withdrew from the study, respectively (Fig. 1).

## Demographic and baseline characteristics

The baseline characteristics between arms were similar and comparable with those reported in the primary analysis (Supplementary Table 3). The representativeness of study participants to the overall population of patients with AML is presented in Supplementary Table 4. The median (range) age of patients was 48.0 (18, 80) years, and 52.9% (146/276) were female. The majority of patients (88.0% [243/276]) were Asian, while the rest were White. Overall, most (76.0% [200/276]) patients had an intermediate cytogenetic risk. Most (88.0% [243/276]) patients had only an *FLT3-*ITD mutation. The rate of prior FLT3 inhibitor use was 12.4% (17/137) and 7.2% (10/139) in the gilteritinib and salvage chemotherapy arms, respectively. Only 2.2% (3/137) of patients in the gilteritinib arm and 4.3% (6/139) of patients in the salvage chemotherapy arm underwent prior HSCT.

## Treatment exposure

In the SAF, 134 patients received gilteritinib, 20 patients received LoDAC, 33 patients received a MEC regimen, and 66 patients received a FLAG regimen. The median (range) duration of exposure was 137.5 (7, 1917) days for the gilteritinib arm and was similar across the 3 salvage chemotherapy regimens (LoDAC, 28.0 [9, 113]; MEC regimen, 28.0 [2, 97]; FLAG regimen, 28.0 [16, 76] days). In the gilteritinib arm, 37.3% (50/134) of patients received at least 1 dose decrease and 29.1% (39/134) of patients received at least 1 dose increase.

## Efficacy endpoints

### Overall survival

**Fig. 2:**
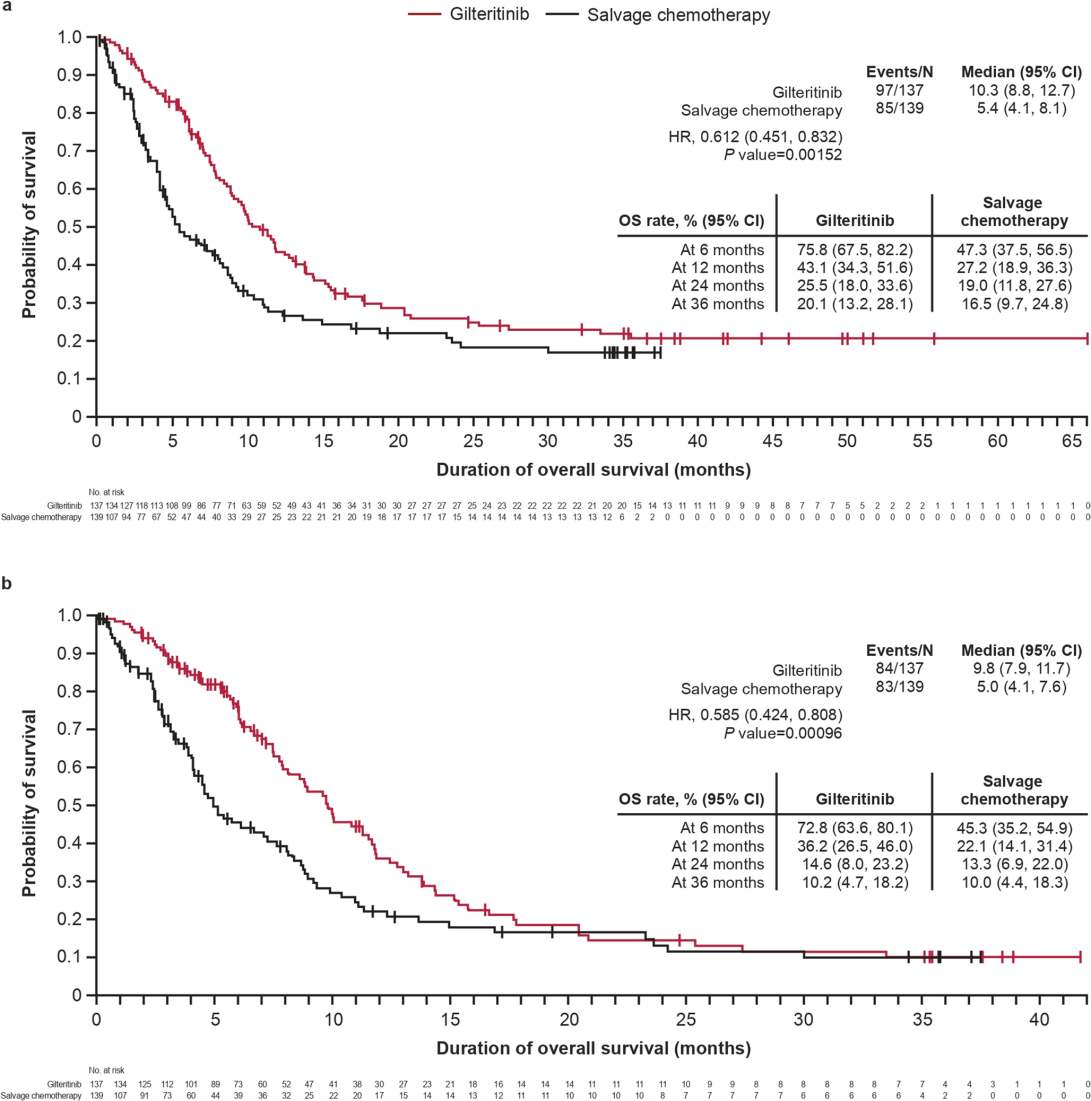
Kaplan-Meier analysis of OS by treatment arm (**a**) and censored at HSCT (**b**). *P* value is from a stratified 2-sided log-rank test. Survival rates and 95% CIs were estimated using the Kaplan-Meier method and Greenwood formula. CI, confidence interval; HR, hazard ratio; HSCT, hematopoietic stem cell transplantation; OS, overall survival

At the end of the study period, 70.8% (97/137) of patients died in the gilteritinib arm and 61.2% (85/139) of patients died in the salvage chemotherapy arm. The median (95% CI) duration of survival follow-up was 37.6 (35.0, 42.0) months in the gilteritinib arm and 34.1 (17.2, 34.4) months in the salvage chemotherapy arm. The median (95% CI) OS was longer in the gilteritinib arm compared with the salvage chemotherapy arm (10.3 [8.8, 12.7] vs 5.4 [4.1, 8.1] months, respectively; HR [95% CI], 0.612 [0.451, 0.832]; Fig. 2a). Of note, fewer patients in the gilteritinib arm (29.2% [40/137]) than the salvage chemotherapy arm (38.8% [54/139]) were censored. At 24 months, the OS rate (95% CI) was 25.5% (18.0, 33.6) in the gilteritinib arm compared with 19.0% (11.8, 27.6) in the salvage chemotherapy arm; at 36 months, the OS rates were 20.1% (13.2, 28.1) and 16.5% (9.7, 24.8) in the gilteritinib and salvage chemotherapy arms, respectively. Similar results were observed when patients were censored at HSCT, and 61.3% (84/137) and 59.7% (83/139) of patients gilteritinib and salvage chemotherapy arms died, respectively (Fig. 2b). The median (95% CI) duration of OS follow-up was 24.7 (13.8, 35.4) months and 17.2 (7.8, 34.4) months in the gilteritinib and salvage chemotherapy arms, respectively.

In patients who achieved CR, median OS was 35.5 months in the gilteritinib arm and 10.3 months in the salvage chemotherapy arm (HR [95%CI], 0.448 (0.200, 1.006); in patients who did not achieve CR, the median OS was 8.8 months in the gilteritinib arm versus 4.7 months in the salvage chemotherapy arm (HR [95% CI], 0.714 [0.521, 0.978]).

**Fig. 3:**
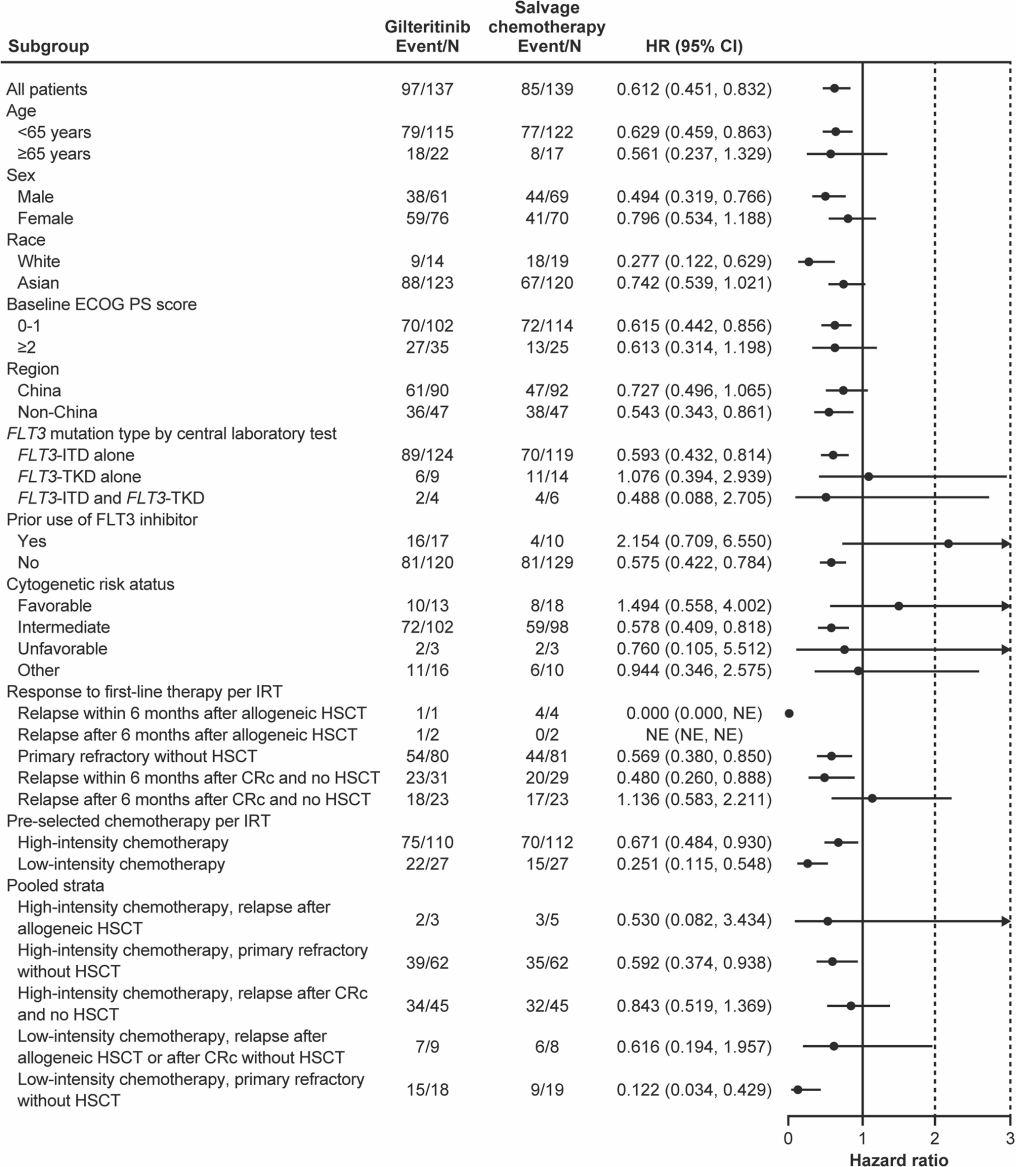
Forest plot of overall survival subgroup analysis. An arrow is displayed for the 95% CI of the HR if the upper bound was greater than 3. An HR and 95% CI for all patients were based on a stratified Cox proportional hazard model with response to first-line AML therapy and preselected salvage chemotherapy per interactive response technology as stratification factors. In each subgroup, the HR and 95% CI were estimated using an unstratified Cox proportional hazard model. Assuming proportional hazards, an HR < 1 indicates a reduction in hazard rate in favor of the gilteritinib arm. AML, acute myeloid leukemia; CI, confidence interval; CRc, composite complete remission; ECOG PS, Eastern Cooperative Oncology Group performance status; FLT3, FMS-like tyrosine kinase 3; HR, hazard ratio; HSCT, hematopoietic stem cell transplantation; ITD, internal tandem duplication; NE, not estimable; TKD, tyrosine kinase domain

The treatment effect of gilteritinib was consistent across most analyzed subgroups (Fig. 3). Of note, median OS was numerically longer for patients in the gilteritinib arm than in the salvage chemotherapy arm in high-intensity preselected chemotherapy (11.5 vs 7.2 months, respectively), low-intensity preselected chemotherapy (8.8 vs 3.9 months), and in patients who were primarily refractory without HSCT after first-line therapy (12.4 vs 5.4 months). Patients who were treated with gilteritinib and had a response to first-line therapy of relapse after 6 months following CRc with no HSCT (7.4 vs 9.9 months) and a favorable cytogenetic risk status at baseline (9.6 vs 14.9 months) did not have numerically longer OS compared with patients who were treated with salvage chemotherapy. However, the number of patients in each subgroup was small.

### Event-free survival

**Fig. 4:**
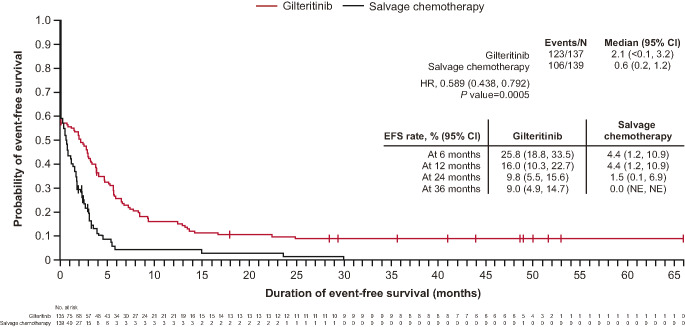
Kaplan-Meier analysis of EFS by treatment arm. *P* value is from a stratified 2-sided log-rank test. EFS rates and 95% CIs were estimated using the Kaplan-Meier method and Greenwood formula. CI, confidence interval; HR, hazard ratio; EFS, event-free survival

The median EFS (95% CI) was numerically longer in the gilteritinib arm (2.1 [< 0.1, 3.2] months) compared with the salvage chemotherapy arm (0.6 [0.2, 1.2] months; HR [95% CI], 0.589 [0.438, 0.792]; Fig. 4). At 24 months, the EFS rate (95% CI) was greater in the gilteritinib arm (9.8% [5.5, 15.6]) compared with the salvage chemotherapy arm (1.5% [0.1, 6.9]); at 36 months, the rates were 9.0% (4.9, 14.7) and 0.0% (not estimable [NE], NE) in the gilteritinib and salvage chemotherapy arms, respectively.

### Complete remission and overall response

**Fig. 5:**
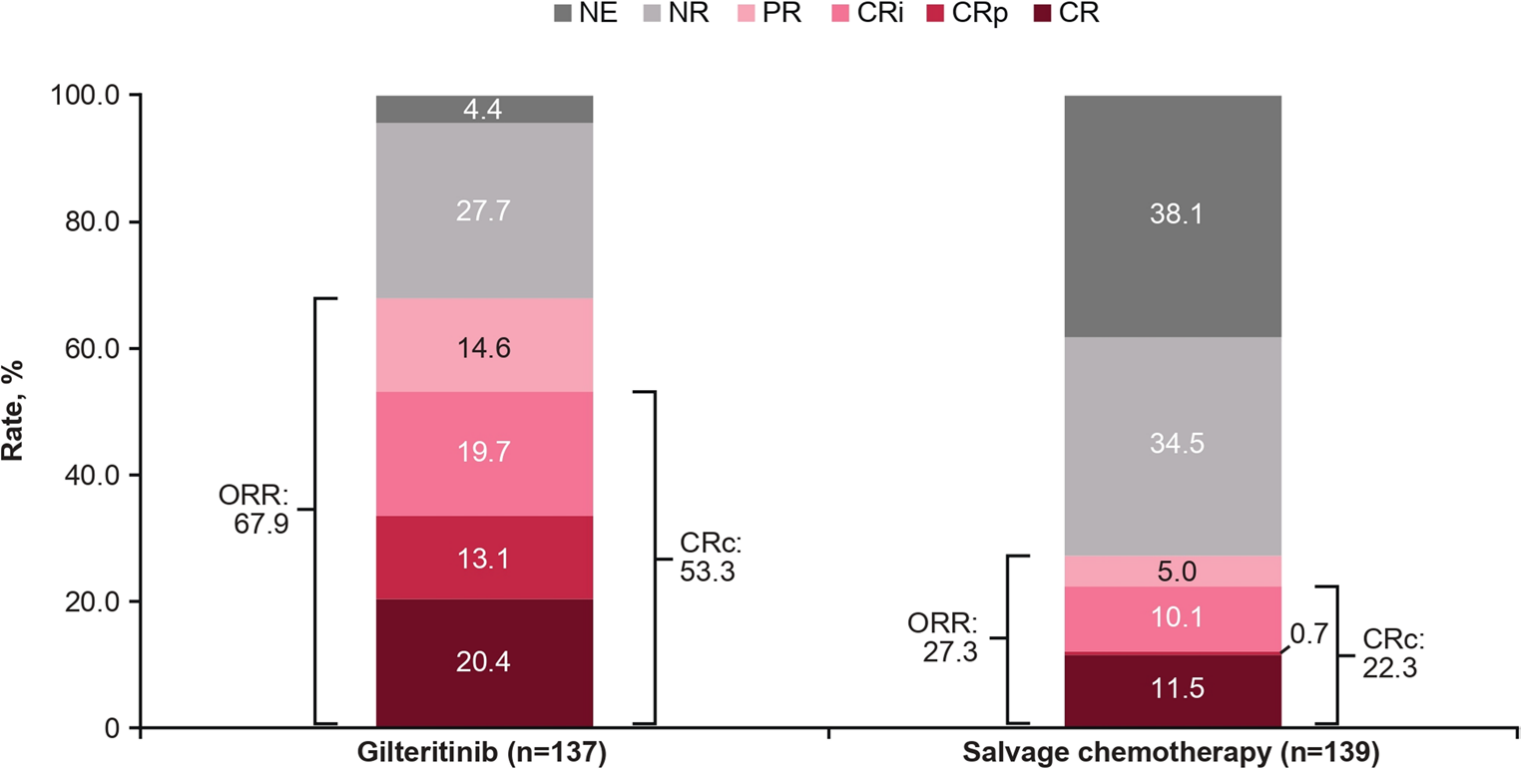
Best overall response. CRc was defined as CR + CRi + CRp. ORR was defined as CRc + PR. All categories were mutually exclusive. CR, complete remission; CRc, composite complete remission; CRi, complete remission with incomplete hematologic recovery; CRp, complete remission with incomplete platelet recovery; NE, not estimable; NR, no response; ORR, overall response rate; PR, partial remission

The CR rate was numerically higher in the gilteritinib arm (20.4%) compared with the salvage chemotherapy arm (11.5%; treatment difference [95% CI], 8.9 [0.3, 17.5]; Fig. 5). The median (95% CI) duration of CR was 24.0 (4.6, NE) months in the gilteritinib arm and NE (1.0, NE) in the salvage chemotherapy arm. The median (range) time to CR was 3.7 (1, 19) months in the gilteritinib arm and 1.1 (1, 2) months in the salvage chemotherapy arm. The CRc rate was 53.3% (73/137) in the gilteritinib arm and 22.3% (31/139) in the salvage chemotherapy arm (treatment difference [95% CI], 31.2 [20.2, 42.3]; Fig. 5). The median duration of CRc was 4.1 (2.8, 5.6) months and NE (NE, NE) in the gilteritinib and salvage chemotherapy arms, respectively. The median (range) time to CRc was 1.8 (1, 8) months in the gilteritinib arm and 1.0 (1, 2) months in the salvage chemotherapy arm. The overall response rate was numerically higher in the gilteritinib arm (67.9% [93/137]) compared with the salvage chemotherapy arm (27.3% [38/139]; Fig. 5). The median time to response (CRc or partial remission) was 1.0 (1, 7) months in the gilteritinib arm and 1.0 (1, 2) months in the salvage chemotherapy arm.

### Transplantation rate and exploratory analyses of survival following gilteritinib resumption

In total, 22.6% (31/137) of patients in the gilteritinib arm and 7.9% (11/139) of patients in the salvage chemotherapy arm underwent HSCT (treatment difference [95% CI], 14.7 [6.5, 22.8]) during the entire study period, which includes subsequent and on-study HSCTs. Only patients in the gilteritinib arm were allowed to undergo on-study HSCT. Of the patients in the gilteritinib arm, 25 patients underwent HSCT without leaving the study.

In a landmark analysis of OS starting from 60 days post-HSCT in patients treated with gilteritinib who underwent HSCT and did not experience relapse within 60 days post-HSCT (*n* = 28), the median (95% CI) OS was NE (28.3, NE) in patients who resumed gilteritinib (*n* = 18) and 15.0 (0.8, NE) months in patients who did not (*n* = 10; HR [95% CI], 0.288 [0.080, 1.034]; Supplementary Fig. 2). The OS rate (95% CI) at 24 months was 77.8% (51.1, 91.0) in patients who resumed gilteritinib versus 30.0% (4.9, 61.8) in patients who did not resume gilteritinib. In patients who resumed gilteritinib (*n* = 18), the median (interquartile range) duration of exposure from 60 days post-HSCT was 888.5 (137.0-1408.0) days.

### Transfusion conversion and maintenance rates

In the gilteritinib arm, 110 patients were dependent on red blood cell or platelet transfusions during the baseline period. In the post-baseline period, 47 patients became transfusion independent, 54 remained transfusion dependent, and 9 were not evaluable. The transfusion conversion rate was 46.5% (47/101). During the baseline period, 24 patients were transfusion independent; of these, 17 patients remained transfusion independent in the post-baseline period and 7 became transfusion dependent post-baseline. The transfusion maintenance rate was 70.8% (17/24).

## Safety

In the SAF, all patients experienced at least 1 treatment-emergent adverse event (TEAE) in both arms. In the gilteritinib arm, 76.1% (102/134), 22.4% (30/134), and 88.8% (119/134) of patients experienced a serious TEAE, a TEAE leading to death, and a drug-related grade 3 or higher TEAE compared with 59.7% (71/119), 13.4% (16/119), and 80.7% (96/119) of patients in the salvage chemotherapy arm, respectively. As patients in the gilteritinib arm had a longer median treatment exposure compared with patients treated with salvage chemotherapy, the rate of serious TEAEs, TEAEs leading to death, and drug-related grade 3 or higher TEAEs (when adjusted by patient-year) was higher in the salvage chemotherapy arm compared with the gilteritinib arm (Table 1). The most frequent drug-related TEAE was anemia in both arms. When adjusted by patient-year, the event rate of drug-related TEAEs was lower in the gilteritinib arm compared with the salvage chemotherapy arm (Supplementary Table 5). The most common serious TEAE was febrile neutropenia in both the gilteritinib (21.6% [29/134]) and salvage chemotherapy (21.0% [25/119]) arms.

**Table 1:** Overview of Treatment-Emergent Adverse Events

	Gilteritinib		Salvage chemotherapy	
	*n* = 134 *n* (%)	PY = 93.3 E (E/PY)	*n* = 119 *n* (%)	PY = 11.7 E (E/PY)
TEAE	134 (100)	9451 (101.3)	119 (100)	4519 (386.2)
Drug-related^a^ TEAE	123 (91.8)	5579 (59.8)	108 (90.8)	3030 (259.0)
Serious TEAE^b^	102 (76.1)	394 (4.2)	71 (59.7)	143 (12.2)
Drug-related^a^ serious TEAE^b^	58 (43.3)	177 (1.9)	45 (37.8)	82 (7.0)
TEAE leading to death	30 (22.4)	41 (0.4)	16 (13.4)	16 (1.4)
Drug-related^a^ TEAE leading to death	12 (9.0)	17 (0.2)	8 (6.7)	8 (0.7)
TEAE leading to withdrawal of treatment	13 (9.7)	23 (0.3)	8 (6.7)	10 (0.9)
Drug-related^a^ TEAE leading to withdrawal of treatment	10 (7.5)	17 (0.2)	6 (5.0)	6 (0.5)
Grade 3 or higher TEAE^c^	131 (97.8)	3367 (36.1)	114 (95.8)	2027 (173.3)
Drug-related^a^ grade 3 or higher TEAE^c^	119 (88.8)	2244 (24.1)	96 (80.7)	1491 (127.4)
TEAE leading to study drug reduction	33 (24.6)	45 (0.5)	1 (0.8)	1 (0.1)
Drug-related^a^ TEAE leading to study drug reduction	29 (21.6)	35 (0.4)	1 (0.8)	1 (0.1)
TEAE leading to study drug interruption	50 (37.3)	127 (1.4)	9 (7.6)	11 (0.9)
Drug-related^a^ TEAE leading to study drug interruption	37 (27.6)	84 (0.9)	8 (6.7)	9 (0.8)

^a^Possible or probable, as assessed by the investigator, or records where the relationship is missing

^b^Includes SAEs upgraded by the sponsor based on review

^c^A missing NCI-CTCAE (v4.03) grade was considered the maximum NCI-CTCAE grade

E, event; NCI-CTCAE, National Cancer Institute–Common Terminology Criteria for Adverse Events; PY, patient-year; SAE, serious adverse event; TEAE, treatment-emergent adverse event

In the gilteritinib arm, AESIs were reported in 94.8% (127/134) of patients in the gilteritinib arm compared with 83.2% (99/119) of patients in the salvage chemotherapy arm. Drug-related grade 3 or higher AESIs were reported by 35.1% (47/134) and 30.3% (36/119) of patients in the gilteritinib and salvage chemotherapy arms, respectively. Differentiation syndrome was the most reported drug-related grade 3 or higher AESI category (gilteritinib, 19.4% [26/134]; salvage chemotherapy, 26.9% [32/119]) and was most commonly febrile neutropenia (gilteritinib, 14.2% [19/134]; salvage chemotherapy, 15.1% [18/119]). There was 1 case of hypoxic-ischemic encephalopathy and prolonged electrocardiogram QT, which were both in the gilteritinib arm. A full list of all drug-related grade 3 or higher AESIs is provided in Supplementary Table 6.

## Discussion

This end-of-study analysis of the COMMODORE trial, with a median follow-up of ~ 3 years, confirmed the survival benefit of gilteritinib compared with salvage chemotherapy in a predominantly Asian population of patients with R/R *FLT3*^mut+^ AML, as previously reported in the interim/primary analysis [9] and regional subgroup analysis [23]. These results further emphasize the long-term benefit of gilteritinib in these patients compared with salvage chemotherapy.

*FLT3*-ITD and *FLT3*-TKD mutations lead to constitutive activation with several downstream signaling pathways that control cell growth and differentiation, which allows leukemic cells to evade normal regulatory mechanisms and expand malignant clones [25]. Thus, sustained inhibition of the FLT3 receptor using a highly selective second-generation inhibitor [12] like gilteritinib can help restore normal hematopoiesis in patients with *FLT3*^mut+^ R/R AML by targeting residual *FLT3*-driven AML clones, which ultimately lowers relapse risk and improves outcomes in this high-risk population [9, 10]. Our results and the available literature support this theory as treatment with gilteritinib led to a significantly improved median OS compared with salvage chemotherapy (10.3 versus 5.4 months, respectively), which is consistent with the COMMODORE trial’s primary analysis [9], the long-term follow-up of the phase 3 ADMIRAL trial [20], and a real-world study with a median follow-up of 21.4 months [26]. Results from other efficacy endpoints, such as EFS, CR, and CRc rates, were also consistent with the COMMODORE trial and the ADMIRAL long-term follow-up [9, 20]. The long-term effect of gilteritinib persisted when compared with salvage chemotherapy, especially in patients who achieved CR. However, the OS rates at 24 and 36 months were low in both arms, highlighting the poor long-term prognosis of patients with R/R *FLT3*^mut+^ AML and that additional treatment strategies are still necessary to improve long-term clinical outcomes.

The effect of gilteritinib on survival was maintained in the majority of analyzed subgroups, similar to the ADMIRAL trial [10]. Of note, the OS subgroup analysis showed that the gilteritinib arm had a statistically longer median OS across R/R patients, except for those who relapsed 6 months after achieving CRc and did not undergo HSCT, compared with the salvage chemotherapy arm. However, these results should be interpreted with caution due to the small number of patients included (ranging from 4-249 patients).

Gilteritinib treatment led to a clear benefit in achieving a response compared with salvage chemotherapy; however, the time to CR was longer in the gilteritinib arm compared with the salvage chemotherapy arm. Of note, the time to response (CRc or partial remission) was similar between arms. The short median duration of chemotherapy exposure (28 days) may have impacted these results. FLT3 inhibition induces terminal myeloid differentiation, which may lead to the appearance of persistent leukemia [27]. As a result, patients treated with gilteritinib may achieve blast clearance after 1 to 3 months [9, 10, 28], but a marrow response, as part of achieving CR, can take several months followed by a full count recovery [28, 29].

Allogeneic HSCT remains the only curative therapy for patients with R/R AML [1]. There is evidence that gilteritinib is an effective bridge to HSCT [22, 30–32]. In the present study, the transplantation rate was greater in the gilteritinib arm compared with the salvage chemotherapy arm, which is in line with what was previously reported in the ADMIRAL trial [20]. Most patients in the gilteritinib arm underwent HSCT during the study period; of these, most resumed gilteritinib after their transplantation.

The clinical benefit of gilteritinib after HSCT remains uncertain. In the present study, a post hoc OS landmark analysis starting from 60 days post-HSCT demonstrated that patients who resumed gilteritinib had longer survival compared with patients who did not. However, small sample sizes and the post hoc nature of this analysis preclude any firm conclusions. Similarly, a post hoc analysis of the ADMIRAL trial found that patients who resumed gilteritinib treatment after HSCT had lower relapse rates and longer OS compared with those who did not, but this analysis was similarly limited [22].

The transfusion conversion rate (46.5%) was broadly similar to what was previously reported [10]. A real-world cohort study has previously reported that over half of patients required transfusion in each of the first 4 cycles of gilteritinib [26]. Here, 7 of 24 patients who were transfusion independent needed transfusions during gilteritinib treatment.

The safety profile of gilteritinib in patients with R/R *FLT3*^mut+^ AML was consistent with the COMMODORE primary analysis [9] and other reports [10, 19]. Similar to the ADMIRAL trial, exposure-adjusted TEAE rates favored gilteritinib versus salvage chemotherapy [20]. Overall, the AESIs reported in the gilteritinib arm were consistent with the known safety profile of gilteritinib [33].

This study was one of the first to look at a predominantly Asian population in a large, prospective, randomized setting over a long-term period. Few patients in this study had prior FLT3 inhibitor use, which may reflect the fact that the study’s enrollment period began before the widespread use of midostaurin [34] and quizartinib [35] during the induction and consolidation phases of patients with *FLT3*^mut+^ AML. However, a retrospective analysis of the CHRYSALIS and ADMIRAL studies demonstrated that previous treatment with FLT3 inhibitors does not affect remission rates in patients with R/R *FLT3*^mut+^ AML [36]. An additional limitation of this study was that measurable residual disease data were not collected. The presence of measurable residual disease is an important prognostic factor for AML [37], and it may have provided additional insights into the effectiveness of gilteritinib in this patient population.

In conclusion, this population of predominantly Asian patients with R/R *FLT3*^mut+^ AML from the COMMODORE study continued to benefit from long-term gilteritinib therapy, further validating the long-term clinical efficacy and safety of gilteritinib treatment.

## Supplementary Information

Below is the link to the electronic supplementary material.

Supplementary Material 1

## Data Availability

Details for how researchers may request access to anonymized participant-level data, trial-level data, and protocols from Astellas-sponsored clinical trials can be found at https://www.clinicaltrials.astellas.com/transparency/.

## Declarations

### Prior presentation

The primary analysis of this study has been previously published (https://doi.org/10.1038/s41375-024-02382-9). These results have been presented in part as an oral presentation at the European Hematology Association Congress from June 12-15, 2025, in Milan, Italy.
