# Supplementary material for: Long-term follow-up of predominantly Asian patients with relapsed/refractory FLT3-mutated acute myeloid leukemia in the phase 3 COMMODORE trial
Source: Ann Hematol. 2026 Jan 12;105(1):3. doi: 10.1007/s00277-026-06762-2 (PMC12791058; doi:10.1007/s00277-026-06762-2)
Supplement: Supplementary file 1 — Supplementary Material 1 [file 277_2026_6762_MOESM1_ESM.docx]

# Supplementary information

## Supplementary Tables

Supplementary Table 1. Response Definitions

| **Response** | **Definition** |
| --- | --- |
| CR | Patients with bone marrow regenerating normal hematopoietic cells and achieving a morphologic leukemia-free state and an ANC ≥1 x 10^9^/L and platelet count ≥100 x 10^9^/L and normal marrow differential with <5% blasts, and RBC and platelet transfusion independent (defined as 1 week without RBC transfusion and 1 week without platelet transfusion).  There should be no evidence of extramedullary leukemia. |
| CRp | Patients fulfilling all criteria for CR except for platelet recovery (platelet count ≥100 x 10^9^/L). |
| CRi | Patients fulfilling all criteria for CR except for hematological recovery (ANC ≥1 x 10^9^/L) with residual neutropenia and complete platelet recovery (platelet count ≥100 x 10^9^/L).  RBC and platelet transfusion independence is not required. |
| CRc | Defined as the remission rate of all CR, CRp, and CRi. |
| PR | Patients with bone marrow regenerating normal hematopoietic cells with evidence of peripheral recovery with no (or only a few regenerating) circulating blasts and with a decrease of at least 50% in the percentage of blasts in the bone marrow aspirate with the total marrow blasts between 5% and 25%. A value of less or equal than 5% blasts is also considered a PR if Auer rods are present. There should be no evidence of extramedullary leukemia. |
| CRh | Patients with post-baseline marrow blasts <5%, partial hematologic recovery ANC ≥0.5 x 10^9^/L and platelets ≥50 x 10^9^/L, no evidence of extramedullary leukemia, and cannot be classified as CR. The blast counts in peripheral blood must be ≤2%. |
| Relapse | Relapse after CR, CRp, or CRi is defined as a reappearance of leukemic blasts in the peripheral blood or ≥5% blasts in the bone marrow aspirate not attributable to any other cause or reappearance or new appearance of extramedullary leukemia.  Relapse after PR is similarly defined with reappearance of significant numbers of peripheral blasts and an increase in the percentage of blasts in the bone marrow aspirate to >25% not attributable to any other cause or reappearance or new appearance of extramedullary leukemia. |
| Best response | Best response is defined as the best measured response to treatment for all visits (in the order of CR, CRp, CRi, PR, NR, and NE) post-treatment. Subjects with best responses of CR, CRp, CRi, or PR will be considered responders. Subjects who do not achieve at least a best response of PR will be considered nonresponders. |

ANC, absolute neutrophil count; CR, complete remission; CRc, composite complete remission; CRh, complete remission with partial hematologic recovery; CRi, complete remission with incomplete hematologic recovery; CRp, complete remission with incomplete platelet recovery; NE, not estimable; NR, no response; PR, partial response; RBC, red blood cell.

Supplementary Table 2. Treatment Disposition

|  | **Gilteritinib  (n=137)** | **Salvage chemotherapy  (n=139)** | **Total (n=276)** |
| --- | --- | --- | --- |
| Ended treatment, n (%) | 137  (100.0) | 139  (100.0) | 276 (100.0) |
| Primary end-of-treatment reason, n (%)^a^ |  |  |  |
| Completed^b^ | 0 | 26  (18.7) | 26  (9.4) |
| Adverse event | 8  (5.8) | 9  (6.5) | 17  (6.2) |
| Death | 19  (13.9) | 8  (5.8) | 27  (9.8) |
| Lack of efficacy | 20  (14.6) | 43  (30.9) | 63  (22.8) |
| Progressive disease | 18  (13.1) | 9  (6.5) | 27  (9.8) |
| Protocol deviation | 4  (2.9) | 1  (0.7) | 5  (1.8) |
| Withdrawal by patient | 10  (7.3) | 28  (20.1) | 38  (13.8) |
| Physician decision | 5  (3.6) | 10  (7.2) | 15  (5.4) |
| Disease relapse | 41  (29.9) | 5  (3.6) | 46  (16.7) |
| Other | 12  (8.8) | 0 | 12  (4.3) |

^a^No patients lost to follow-up or ended treatment due to noncompliance with the study drug or pregnancy.

^b^Refers to patients on high intensity chemotherapy who either completed 1 cycle of treatment with a CRc and were taken off treatment or completed 2 cycles of treatment.

CRC, composite complete remission.

Supplementary Table 3. Demographic and Baseline Characteristics

|  | **Gilteritinib (N=137)** | **Salvage chemotherapy (N=139)** | **Total (N=276)** |
| --- | --- | --- | --- |
| Female, n (%) | 76 (55.5) | 70 (50.4) | 146 (52.9) |
| Race, n (%) |  |  |  |
| Asian | 123 (89.8) | 120 (86.3) | 243 (88.0) |
| White | 14 (10.2) | 19 (13.7) | 33 (12.0) |
| Median (range) age, years | 50.0 (18, 80) | 47.0 (18, 74) | 48.0 (18, 80) |
| Region, n (%) |  |  |  |
| China | 90 (65.7) | 92 (66.2) | 182 (65.9) |
| Baseline ECOG group, n (%) |  |  |  |
| 0-1 | 102 (74.5) | 114 (82.0) | 216 (78.3) |
| ≥2 | 35 (25.5) | 25 (18.0) | 60 (21.7) |
| FLT3 mutation type by central laboratory testing, n (%) |  |  |  |
| *FLT3*-ITD | 124 (90.5) | 119 (85.6) | 243 (88.0) |
| *FLT3*-TKD | 9 (6.6) | 14 (10.1) | 23 (8.3) |
| *FLT3*-ITD and *FLT3*-TKD | 4 (2.9) | 6 (4.3) | 23 (8.3) |
| Prior use of FLT3 inhibitor, n (%)^a^ | 17 (12.4) | 10 (7.2) | 27 (9.8) |
| Cytogenetic risk status, n (%)^b^ | n=134 | n=129 | n=263 |
| Favorable | 13 (9.7) | 18 (14.0) | 31 (11.8) |
| Intermediate | 102 (76.1) | 98 (76.0) | 200 (76.0) |
| Unfavorable | 3 (2.2) | 3 (2.3) | 6 (2.3) |
| Other | 16 (11.9) | 10 (7.8) | 26 (9.9) |
| Prior HSCT, n (%) | 3 (2.2) | 6 (4.3) | 9 (3.3) |
| *NPM1* co-mutation, n (%)^c^ | 23 (16.8)^d^ | 34 (24.5) | 57 (20.7)^d^ |
| Response to first-line therapy per CRF, n (%) |  |  |  |
| Relapse within 6 months after allogeneic HSCT | 0 | 3 (2.3) | 3 (1.1) |
| Relapse after 6 months after allogeneic HSCT | 3 (2.2) | 3 (2.2) | 6 (2.2) |
| Primary refractory without HSCT | 79 (57.7) | 80 (57.6) | 159 (57.6) |
| Relapse within 6 months after CRc and no HSCT | 31 (22.6) | 28 (20.1) | 59 (21.4) |
| Relapse after 6 months after CRc and no HSCT | 24 (17.5) | 25 (18.0) | 49 (17.8) |
| Type of treatment, n (%) |  |  |  |
| Induction | 137 (100.0) | 138 (99.3) | 275 (99.6) |
| Consolidation | 43 (31.4) | 51 (36.7) | 94 (34.1) |
| Maintenance | 16 (11.7) | 12 (8.6) | 28 (10.1) |
| Salvage | 1 (0.7) | 3 (2.2) | 4 (1.4) |
| Prior induction therapy, n (%) | n=137 | n=138 | n=275 |
| Standard dose cytarabine + daunorubicin | 34 (24.8) | 40 (29.0) | 74 (26.9) |
| Standard dose cytarabine + daunorubicin and other^¶^ | 12 (8.8) | 13 (9.4) | 25 (9.1) |
| Standard dose cytarabine + idarubicin | 10 (7.3) | 11 (8.0) | 21 (7.6) |
| Standard dose cytarabine + mitoxantrone | 3 (2.2) | 5 (3.6) | 8 (2.9) |
| Standard dose cytarabine + mitoxantrone and other^e^ | 4 (2.9) | 1 (0.7) | 5 (1.8) |
| Standard dose cytarabine + idarubicin and other^e^ | 2 (1.5) | 3 (2.2) | 5 (1.8) |
| Standard dose cytarabine + daunorubicin and standard dose cytarabine + mitoxantrone | 3 (2.2) | 2 (1.4) | 5 (1.8) |
| High-dose cytarabine + daunorubicin | 1 (0.7) | 0 | 1 (0.4) |
| Low-dose cytarabine and other^e^ | 1 (0.7) | 1 (0.7) | 2 (0.7) |
| High-dose cytarabine and standard dose cytarabine + mitoxantrone | 0 | 1 (0.7) | 1 (0.4) |
| Low-dose cytarabine and decitabine | 0 | 1 (0.7) | 1 (0.4) |
| Low-dose cytarabine, decitabine and other^e^ | 1 (0.7) | 0 | 1 (0.4) |
| Azacitidine and other^e^ | 1 (0.7) | 0 | 1 (0.4) |
| Decitabine and other^e^ | 0 | 1 (0.7) | 1 (0.4) |
| Other^e^ | 63 (46.0) | 58 (42.0) | 121 (44.0) |

^a^Included patients who received prior AML therapy of midostaurin, sorafenib or quizartinib.

^b^The worst category includes those who had multiple risk status. The category of “other” includes those with cytogenetic risk status that cannot be categorized as favorable, intermediate or unfavorable. The denominator was the number of patients whose cytogenetic risk status was available.

^c^As per WHO classification.

^d^One patient had AML with t(8;21)(q22;q22); RUNX1-RUNX1T1 and one patient had AML with mutated *NPM1* and AML with maturation.

^e^Other than standard dose cytarabine + idarubicin, standard dose cytarabine + daunorubicin, standard dose cytarabine + daunorubicin + cladribine, high-dose cytarabine + idarubicin, high-dose cytarabine + daunorubicin, high-dose cytarabine, standard dose cytarabine + mitoxantrone, low-dose cytarabine, azacitidine and decitabine.

AML, acute myeloid leukemia; CRc, composite complete remission; CRF, case report form; ECOG, Eastern Cooperative Oncology Group; *FLT3*, *FMS*-like tyrosine kinase 3; HSCT, hematopoietic stem cell transplantation; ITD, internal tandem duplication; *NPM1*, nucleophosmin 1; RUNX1, runt-related transcription factor 1; RUNX1T1, runt-related transcription factor 1; translocated to 1; TKD, tyrosine kinase domain; WHO, World Health Organization.

**Supplementary Table 4.** Representativeness of Study Participants

| **Cancer type(s)/subtype(s)/stage(s)/condition** | **Relapsed/refractory *FLT3*-mutated (R/R *FLT3*^mut+^) acute myeloid leukemia (AML)** |
| --- | --- |
| Other considerations | In 2021 the global age-standardized incidence of AML was 1.73 per 100,000 persons. In patients with newly diagnosed AML, *FLT3-*internal tandem duplications (*FLT3*-ITD) and point mutations in the tyrosine kinase domain (*FLT3*-TKD) occur in 18.9-24.9% and 5.0-6.2% of newly diagnosed adult patients with AML, respectively. Relapse is common in AML with more than 50% of patients relapsing after first-line treatment, while up to 30% of patients are refractory to treatment. |
| Considerations related to: | |
| Sex | In 2017, the global age-standardized incidence of AML in males was 8.1 per 100,000 persons and 5.6 per 100,000 persons in females. |
| Age | From 2018 to 2022, in the United States, AML is most frequently diagnosed among people aged 65-74 years, with a median age at diagnosis of 69 years.  In 2017, the global age-standardized incidence of AML in patients 50 to 69 years of age was 11.4 per 100,000 persons. |
| Race/ethnicity | In the United States from 2018 to 2022, the overall AML incidence rate was 4.1 cases per 100,00 persons among Hispanic men, 4.2 cases per 100,000 persons in Asian/Pacific Islander men, 4.3 cases per 100,000 persons in Black men, and 5.5 cases per 100,000 persons in White men.  In women, the incidence rates were 3.3, 3.0, 3.5, and 3.8 per 100,000 persons in Hispanic, Asian/Pacific Islander, Black, and White women, respectively. |
| Geography | In 2021, the age-standardized incidence of AML was 2.13 per 100,000 persons and 1.0 per 100,000 persons in Southeast Asia and China, respectively. In 2021 the age-standardized incidence of AML was 1.4 per 100,000 persons in Russia. |
| Overall representativeness of this study | The median age of participants in our study (48.0 years) is lower than the median age of AML diagnosis of 69 years in the United States.  The incidence of AML is greater in males compared with females, but this study included a similar number of female and male patients.  Our study had a predominantly Asian patient population due to the study protocol with recruitment sites in China, Southeast Asia, and Russia. However, this was one of the aims of the phase 3 clinical trial, as the efficacy and safety of gilteritinib in patients with R/R *FLT3*^mut+^ AML has already been demonstrated in a predominantly White patient population in the phase 3 ADMIRAL trial (Perl *et al*., 2019; DOI: 10.1056/NEJMoa1902688).  Furthermore, only 12.0% of patients were White and no patients were Black or Hispanic, but this is likely because of the study being restricted to sites in Asia. |

AML, acute myeloid leukemia; *FLT3*, *FMS*-like tyrosine kinase 3; *FLT3*^mut+^, *FLT3*-mutated; ITD, internal tandem duplication; R/R, relapsed/refractory; TKD, tyrosine kinase domain.

Supplementary Table 5. Most Frequent^a^ Drug-Related TEAEs

| **Preferred term** | **Gilteritinib** | **Salvage chemotherapy** |
| --- | --- | --- |

|  | **All** | | **≥Grade 3** | | **All** | | **≥Grade 3** | |
| --- | --- | --- | --- | --- | --- | --- | --- | --- |
|  | **n=134 n (%)** | **PY=93.3 E (E/PY)** | **n=134 n (%)** | **PY=93.3 E (E/PY)** | **n=119 n (%)** | **PY=11.7 E (E/PY)** | **n=119 n (%)** | **PY=11.7 E (E/PY)** |

| Overall | 123 (91.8) | 5579 (59.8) | 119 (88.8) | 2244 (24.1) | 108 (90.8) | 3030 (259.0) | 96 (80.7) | 1491 (127.4) |
| --- | --- | --- | --- | --- | --- | --- | --- | --- |
| Anemia | 80 (59.7) | 518 (5.6) | 69 (51.5) | 231 (2.5) | 73 (61.3) | 328 (28.0) | 68 (57.1) | 170 (14.5) |
| Decreased white blood cell count | 54 (40.3) | 485 (5.2) | 50 (37.3) | 271 (2.9) | 47 (39.5) | 201 (17.2) | 47 (39.5) | 139 (11.9) |
| Decreased platelet count | 53 (39.6) | 614 (6.6) | 50 (37.3) | 440 (4.7) | 48 (40.3) | 377 (32.2) | 48 (40.3) | 311 (26.6) |
| Decreased neutrophil count | 49 (36.6) | 478 (5.1) | 47 (35.1) | 313 (3.4) | 42 (35.3) | 194 (16.6) | 40 (33.6) | 119 (10.2) |
| Thrombocytopenia | 44 (32.8) | 450 (4.8) | 38 (28.4) | 288 (3.1) | 36 (30.3) | 243 (20.8) | 36 (30.3) | 195 (16.7) |
| Increased aspartate aminotransferase | 42 (31.3) | 117 (1.3) | 2 (1.5) | 4 (<0.1) | 11 (9.2) | 19 (1.6) | 1 (0.8) | 1 (0.1) |
| Neutropenia | 39 (29.1) | 350 (3.8) | 34 (25.4) | 174 (1.9) | 22 (18.5) | 75 (6.4) | 22 (18.5) | 51 (4.4) |
| Increased alanine aminotransferase | 37 (27.6) | 123 (1.3) | 7 (5.2) | 10 (0.1) | 13 (10.9) | 25 (2.1) | 1 (0.8) | 3 (0.3) |
| Increased blood lactate dehydrogenase | 37 (27.6) | 66 (0.7) | 3 (2.2) | 5 (0.1) | 12 (10.1) | 19 (1.6) | 0 | 0 |
| Leukopenia | 33 (24.6) | 350 (3.8) | 32 (23.9) | 156 (1.7) | 29 (24.4) | 143 (12.2) | 29 (24.4) | 93 (8.0) |
| Increased blood creatine phosphokinase | 27 (20.1) | 62 (0.7) | 3 (2.2) | 4 (<0.1) | 1 (0.8) | 4 (0.3) | 1 (0.8) | 1 (0.1) |
| Decreased lymphocyte count | 23 (17.2) | 205 (2.2) | 16 (11.9) | 76 (0.8) | 24 (20.2) | 134 (11.5) | 21 (17.6) | 94 (8.0) |
| Hypokalemia | 21 (15.7) | 42 (0.5) | 6 (4.5) | 9 (0.1) | 22 (18.5) | 57 (4.9) | 5 (4.2) | 9 (0.8) |
| Pyrexia | 21 (15.7) | 29 (0.3) | 4 (3.0) | 4 (<0.1) | 23 (19.3) | 47 (4.0) | 3 (2.5) | 4 (0.3) |
| Increased blood alkaline phosphatase | 19 (14.2) | 50 (0.5) | 0 | 0 | 5 (4.2) | 14 (1.2) | 0 | 0 |
| Febrile neutropenia | 19 (14.2) | 28 (0.3) | 19 (14.2) | 28 (0.3) | 18 (15.1) | 23 (2.0) | 18 (15.1) | 23 (2.0) |
| Hyperglycemia | 19 (14.2) | 33 (0.4) | 1 (0.7) | 1 (<0.1) | 12 (10.1) | 22 (1.9) | 1 (0.8) | 1 (0.1) |
| Increased gamma-glutamyltransferase | 18 (13.4) | 64 (0.7) | 5 (3.7) | 8 (0.1) | 8 (6.7) | 19 (1.6) | 1 (0.8) | 1 (0.1) |
| Increased blood bilirubin | 17 (12.7) | 32 (0.3) | 3 (2.2) | 3 (<0.1) | 8 (6.7) | 22 (1.9) | 1 (0.8) | 1 (0.1) |
| Hypocalcemia | 17 (12.7) | 66 (0.7) | 3 (2.2) | 8 (0.1) | 14 (11.8) | 41 (3.5) | 2 (1.7) | 4 (0.3) |
| Pneumonia | 17 (12.7) | 30 (0.3) | 12 (9.0) | 15 (0.2) | 15 (12.6) | 19 (1.6) | 12 (10.1) | 12 (1.0) |
| Diarrhea | 16 (11.9) | 19 (0.2) | 1 (0.7) | 1 (<0.1) | 14 (11.8) | 16 (1.4) | 1 (0.8) | 1 (0.1) |
| Upper respiratory tract infection | 15 (11.2) | 22 (0.2) | 7 (5.2) | 9 (0.1) | 2 (1.7) | 2 (0.2) | 0 | 0 |
| Hyponatremia | 14 (10.4) | 31 (0.3) | 4 (3.0) | 5 (0.1) | 12 (10.1) | 40 (3.4) | 3 (2.5) | 6 (0.5) |

^a^Drug-related TEAEs that occurred in ≥10.0% of patients in the gilteritinib arm.

Adverse events were summarized by the MedDRA v23.0.

E, events; PY, patient-year; TEAE, treatment-emergent adverse event.

Supplementary Table 6. Drug-related Grade 3 or higher AESIs

| **AESI category**  **Preferred term** | **Gilteritinib (n=134) n (%)** | **Salvage chemotherapy (n=119) n (%)** |
| --- | --- | --- |
| **Overall** | 47 (35.1) | 36 (30.3) |
| **Differentiation syndrome^a^** | 26 (19.4) | 32 (26.9) |
| Febrile neutropenia | 19 (14.2) | 18 (15.1) |
| Pneumonia | 6 (4.5) | 11 (9.2) |
| Pyrexia | 3 (2.2) | 3 (2.5) |
| Dyspnea | 1 (0.7) | 0 |
| Acute lung injury | 0 | 1 (0.8) |
| Acute respiratory failure | 0 | 1 (0.8) |
| Cough | 0 | 1 (0.8) |
| Multiple organ dysfunction syndrome | 0 | 1 (0.8) |
| Respiratory failure | 0 | 1 (0.8) |
| **Increased liver transaminase** | 16 (11.9) | 4 (3.4) |
| Increased alanine aminotransferase | 7 (5.2) | 1 (0.8) |
| Increased gamma-glutamyltransferase | 5 (3.7) | 1 (0.8) |
| Increased blood bilirubin | 3 (2.2) | 1 (0.8) |
| Increased aspartate aminotransferase | 2 (1.5) | 1 (0.8) |
| Abnormal hepatic function | 2 (1.5) | 1 (0.8) |
| Increased conjugated bilirubin | 1 (0.7) | 0 |
| Increased transaminases | 1 (0.7) | 0 |
| **Increased creatine phosphokinase** | 6 (4.5) | 1 (0.8) |
| Increased blood creatine phosphokinase | 3 (2.2) | 1 (0.8) |
| Myalgia | 1 (0.7) | 0 |
| Increased myoglobin blood | 1 (0.7) | 0 |
| Rhabdomyolysis | 1 (0.7) | 0 |
| **Pancreatitis** | 4 (3.0) | 2 (1.7) |
| Increased blood bilirubin | 3 (2.2) | 1 (0.8) |
| Increased amylase | 1 (0.7) | 0 |
| Vomiting | 0 | 1 (0.8) |
| **Cardiac failure** | 3 (2.2) | 1 (0.8) |
| Cardiac failure | 3 (2.2) | 1 (0.8) |
| **Posterior reversible encephalopathy syndrome** | 1 (0.7) | 0 |
| Hypoxic-ischemic encephalopathy | 1 (0.7) | 0 |
| **QT prolongation** | 1 (0.7) | 0 |
| Prolonged electrocardiogram QT | 1 (0.7) | 0 |
| **Anaphylactic reaction** | 2 (1.5) | 3 (2.5) |
| Acute respiratory failure | 0 | 1 (0.8) |
| Cough | 0 | 1 (0.8) |
| Dyspnoea | 1 (0.7) | 0 |
| Rash | 1 (0.7) | 0 |
| Respiratory failure | 0 | 1 (0.8) |
| Pharyngeal swelling | 0 | 1 (0.8) |
| **Gastrointestinal obstruction** | 0 | 1 (0.8) |
| Small intestinal obstruction | 0 | 1 (0.8) |
| **Gastrointestinal perforation** | 0 | 1 (0.8) |
| Anal abscess | 0 | 1 (0.8) |

^a^Differentiation syndrome was only summarized for TEAEs which occurred within the first 90 days of treatment.

AESI, adverse events of special safety interest; TEAE, treatment-emergent adverse event.

## Supplementary Figures

Supplementary Fig. 1 Study design


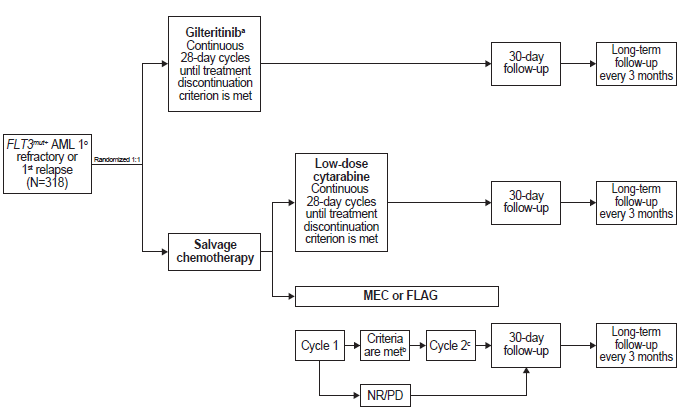


^a^Patients who had a donor identified and achieved a response allowing them to undergo HSCT could have undergone HSCT without leaving the study. However, gilteritinib was stopped and patients underwent a pre-HSCT visit before starting the conditioning regimen for HSCT. Gilteritinib could have been resumed after HSCT if: patient was between 30-90 days after HSCT; patient had successful engraftment as demonstrated by ANC ≥500/mm^3^ and platelets ≥2000/mm^3^ without transfusions; patients did not have ≥grade 2 acute graft versus host disease; and patient was in composite complete remission.

^b^On or after Day 15, if the bone marrow cellularity was 20% or greater with at least a 50% reduction in blasts, the patient may have received a second cycle of the same chemotherapy. If bone marrow cellularity was between 5% and 20%, the investigator made the decision whether the patient should have received another treatment cycle or should have been observed for recovery. If bone marrow cellularity was 5% or less, the patient was observed for recovery.

^c^Patients achieving CR, CRi or CRp may have received a second cycle of chemotherapy at the investigator’s discretion. Patients with NR or progressive disease following Cycle 1 discontinued study treatment.

1º, primary; ANC, absolute neutrophil count; AML, acute myeloid leukemia; CR, complete remission; CRi, complete remission with incomplete hematologic recovery; CRp, complete remission with incomplete platelet recovery; FLAG, fludarabine, high-dose cytarabine, and granulocyte colony-stimulating factor; *FLT3*, *FMS-*like tyrosine kinase *3*; *FLT3*^mut+^, *FLT3*-mutated; HSCT, hematopoietic stem cell transplant; MEC, mitoxantrone, etoposide, and intermediate-dose cytarabine; NR, no response; PD, progressive disease.

**Supplementary Fig. 2** Overall survival landmark analysis starting from 60 days post-HSCT by treatment resumption in patients in the gilteritinib arm who underwent HSCT and did not experience relapse within 60 days post-HSCT

**
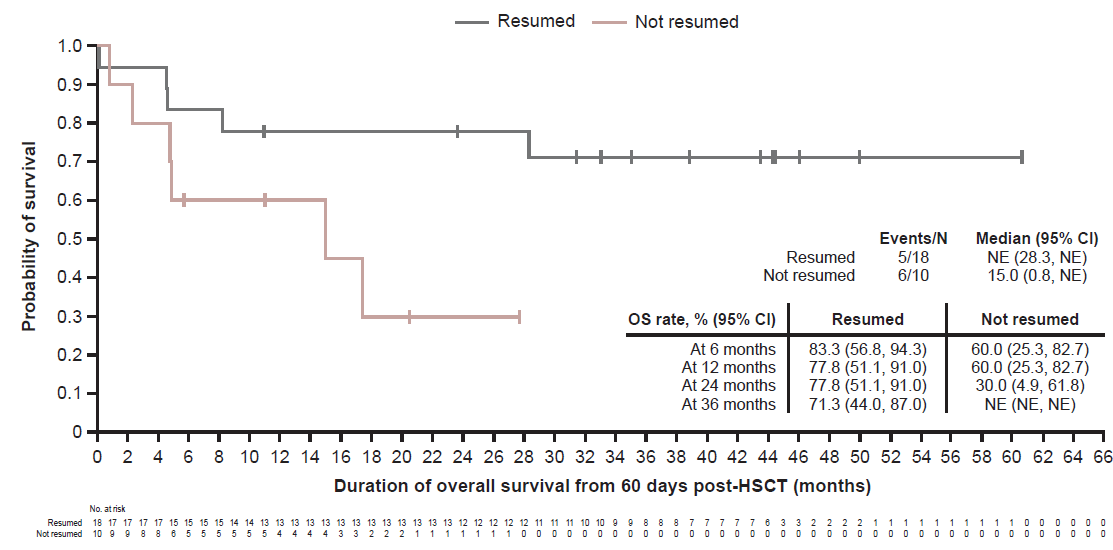
**

Survival rates and 95% CI were estimated using Kaplan-Meier Method and Greenwood formula.

CI, confidence interval; HSCT, hematopoietic stem cell transplantation; NE, not estimable.
